# *Helicobacter pylori* VacA induces autophagic cell death in gastric epithelial cells via the endoplasmic reticulum stress pathway

**DOI:** 10.1038/s41419-017-0011-x

**Published:** 2017-12-13

**Authors:** Pan Zhu, Jun Xue, Zhu-jun Zhang, Yin-ping Jia, Ya-nan Tong, Dan Han, Qian Li, Yang Xiang, Xu-hu Mao, Bin Tang

**Affiliations:** 1 Department of Clinical Microbiology and Immunology, Southwest Hospital and College of Medical Laboratory Science, Third Military Medical University, Chongqing, 400038 China; 2 Emei Sanatorium of PLA Rocket Force, Emeishan, 614205 China

## Abstract

The *Helicobacter pylori* vacuolating cytotoxin (VacA) can promote progressive vacuolation and gastric injury and may be associated with human gastric cancer. Increasing evidence indicates that autophagy is involved in the cell death induced by VacA, but the specific mechanisms need to be further elucidated. We show here that VacA could induce autophagy and increase cell death in human gastric cancer cell lines. Further investigations revealed that inhibition of autophagy could decrease the VacA-induced cell death in AGS cells. Furthermore, numerous dilated endoplasmic reticula (ER) were observed, and the phosphorylation of a subunit of eukaryotic translation initiation factor 2 subunit 1 also increased in the VacA-treated AGS cells, while repression of ER stress could reduce autophagy and cell death through knockdown of activating transcription factor 4 and DNA-damage-inducible transcript 3. In addition, the expression of pseudokinase tribbles homolog 3 (TRIB3) upon ER stress was triggered by VacA, and knockdown of TRIB3 could also decrease VacA-induced cell death. Finally, inhibition of autophagy could decrease VacA^*s1m1*^-induced cell death and apoptosis, and apoptosis inhibitor Z-VAD had no significant effect on autophagy induced by VacA^*s1m1*^. Thus, these results suggested that VacA causes autophagic cell death via ER stress in gastric epithelial cells.

## Introduction

*Helicobacter pylori* (*H. pylori*) is a Gram-negative bacterium colonizing the human stomach, and the prevalence of its infection in adults is 50–90% in developing countries and between 30 and 50% in developed countries^1^. *H. pylori-*persistent infection can contribute to gastritis and peptic ulcer disease and is a definite pathogenic factor for gastric adenocarcinoma^2–4^. The interactions among the bacterium, gastric epithelium, and host innate defense responses are critical for the consequences of *H. pylori* infection. Vacuolating cytotoxin (VacA), a critical virulence factor of *H. pylori*, is considered a multifunctional toxin, responsible for eliciting several different effects on host cells, including vacuolization, necrosis, and apoptosis^5–7^. Numerous studies have shown that VacA can induce apoptosis through the mitochondrial pathway in gastric epithelial cells^7–9^. Alternatively, most VacA was localized to vacuoles rather than mitochondria using immunostaining and confocal microscopy with labeled Rab7-GFP^10^, indicating that VacA may indirectly lead to cytochrome *c* release from mitochondria, which suggests that VacA may involve other pathways leading to cell death.

The endoplasmic reticulum (ER) is a complex, multifunctional organelle that has a critical role in cellular biological effects by synthesizing proteins and monitoring protein folding and trafficking^11,12^. If the ER cannot resolve cell stress, it will cause unfolded or misfolded proteins to accumulate in the ER lumen, leading to ER stress, which is involved in signaling pathways, including inflammation and cell death^13^. To guard against or respond to ER stress, cells develop an integrated signaling mechanism to restore homeostasis and normal ER function^14^. ER stress activates a series of downstream transcriptional effectors, such as nuclear protein 1 (NUPR1), eukaryotic translation initiation factor 2 subunit 1 (EIF2S1), activating transcription factor 4 (ATF4), DNA-damage-inducible transcript 3 (DDIT3), and tribbles pseudokinase 3 (TRIB3), to regulate protein folding and protein quality control^15^. The coordination activity of the entire process determines the extent of endoplasmic reticulum stress and thus governs whether cells will re-establish an intracellular biological balance or activate cell death programs.

Macroautophagy (hereafter autophagy) is an intracellular quality-control and quantity-control process in which intracellular components are sequestered into double-membrane organelles and are delivered to lysosomes for degradation^16^. In addition to the protective role of cell homeostasis, including nutrient starvation and hypoxia stress, prolonged autophagy or overstimulated autophagy could contribute to autophagic cell death^17,18^. Recently, we showed that Shiga toxins purified from *Escherichia coli O157:H7* result in autophagic cell death in Caco-2 cells through the ER stress signaling pathway^17^. In addition, gene products from other bacteria have been reported to participate in autophagic cell death^19,20^. The “enhanced intracellular survival” (eis) gene product of *Mycobacterium tuberculosis* can regulate inflammation and lead to autophagic cell death through redox-dependent signaling in macrophages^21^. Although some studies have reported that VacA of *H. pylori* can induce autophagy, the mechanism by which VacA induces cell death remains to be elucidated.

In this study, the relationships among VacA, ER stress, autophagy, and cell death were investigated in AGS cells. We provide evidence showing that *H. pylori* VacA induces autophagic cell death in gastric epithelial cells through the ER stress pathway.

## Results

### VacA induces cell death in human gastric cancer cells

**Fig. 1:**
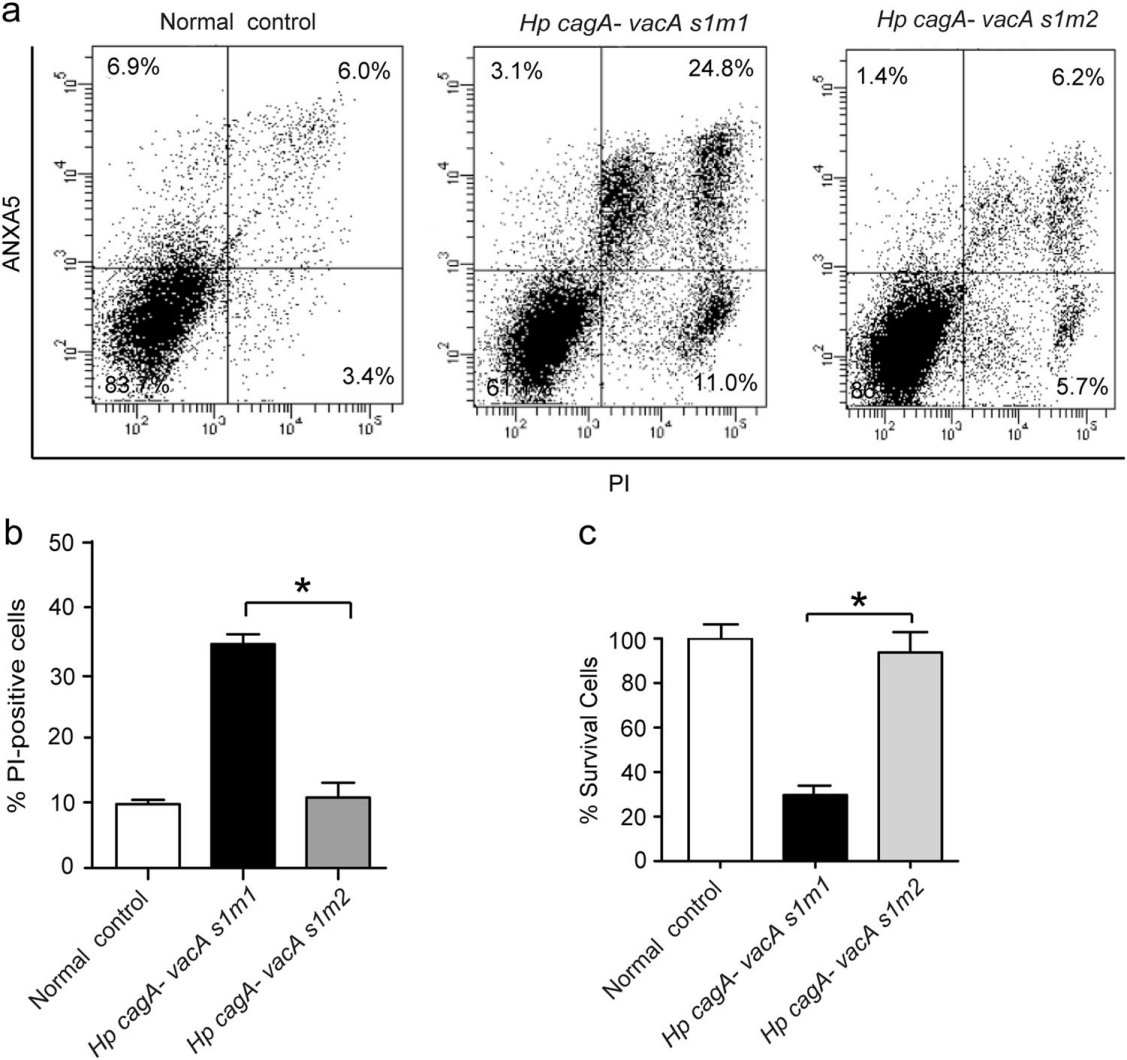
VacA induces cell death in human gastric cancer cells (**a**) AGS cells were infected with *H. pylori cagA* ^−^ *VacA* ^*s1m1*^, *H. pylori cagA* ^−^ *VacA* ^*s1m2*^ and the control (MOI = 100:1) for 24 h; the cells were then subjected to ANXA5-PI staining and analyzed by flow cytometry. (**b**) The percentage of cells that were PI-positive relative to the total cell number for each treatment is shown. (**c**) AGS cells were treated with the indicated bacteria for 24 h. Cell viability was assessed using an MTT assay. The data are presented as the mean ± SEM of three independent experiments. *P < 0.05

Previous studies have indicated that VacA rapidly induces apoptosis and programmed cell necrosis of gastric cancer cells^6,22^. To determine whether VacA was associated with cell death, we employed an ANXA5/propidium iodide (PI) staining assay to detect AGS cells infected with *cagA* ^−^/*vacA* ^*s1m1*^ and *cagA* ^−^/*vacA* ^*s1m2*^ *H. pylori*. The *cagA* ^−^/*vacA* ^*s1m1*^ *H. pylori* infection markedly increased cell death compared with *cagA* ^−^/*vacA* ^*s1m2*^ *H. pylori* (Figs. 1a, b). To further investigate the level of cell death induced by VacA, we performed an MTT assay. Similar results were also obtained in AGS cells infected with *cagA* ^−^/*vacA* ^*s1m1*^ and *cagA* ^−^/*vacA* ^*s1m2*^ *H. pylori* (Fig. 1c). These data indicate that VacA has a critical role in *H. pylori*-induced cell death in human gastric cancer cells.

### Cell death induced by VacA is dependent on autophagy

**Fig. 2:**
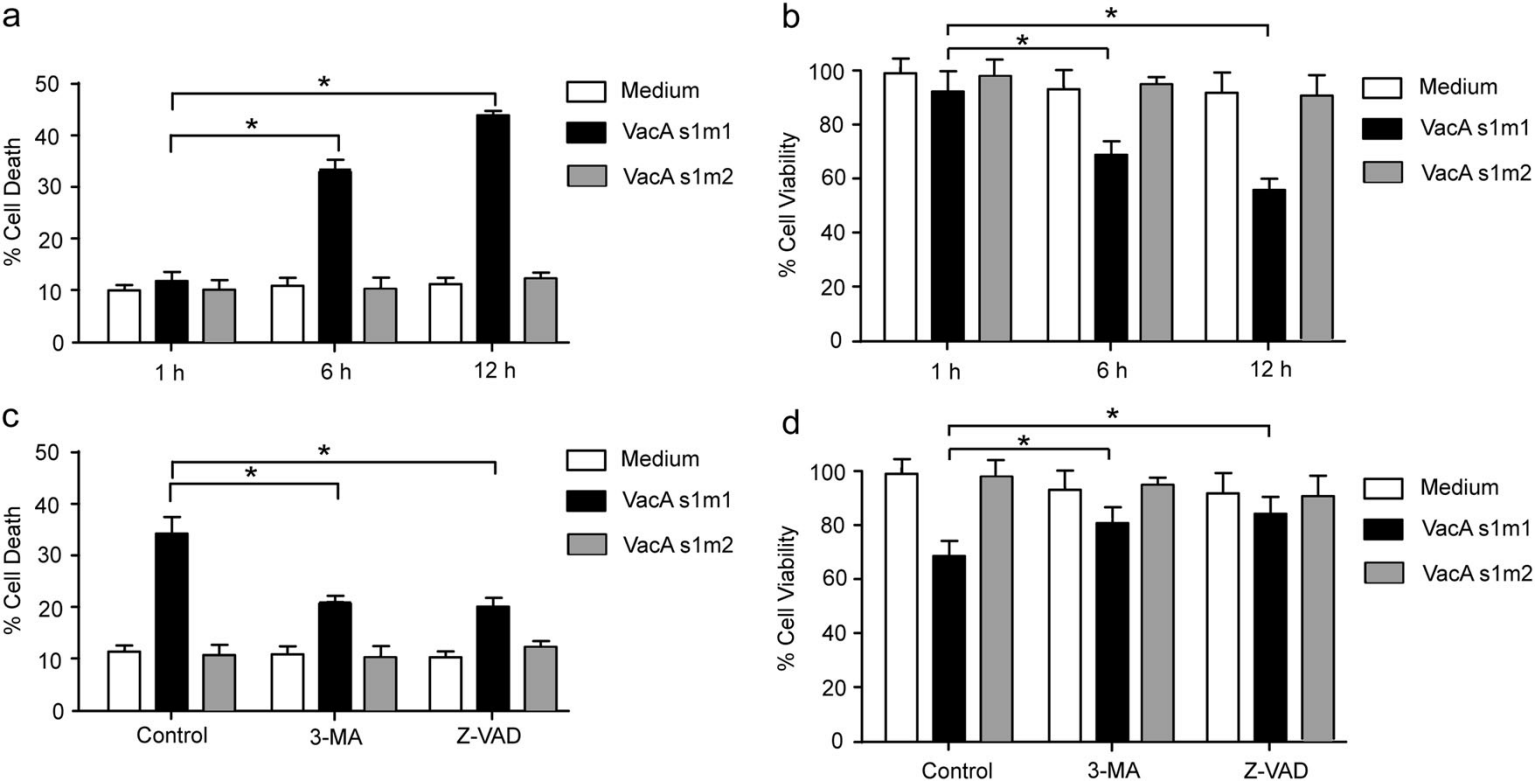
Cell death induced by VacA is dependent on autophagy (**a**, **b**) VacA^*s1m1*^ induced cell death in AGS cells. (**c**, **d**) The effects of 3-MA or Z-VAD on the cytotoxicity of VacA in AGS cells. After pretreatment with 2 mM 3-MA or 50 mM Z-VAD, AGS cells were treated with VacA^*s1m1/ s1m2*^ toxin for 6 h. The percentage of dead cells was determined using the cell death assay (PI staining) or the cell viability assay (MTT). The results shown are representative of at least three independent experiments. *P < 0.05

To further investigate the role of VacA in the *H. pylori*-induced human gastric epithelial cell death, AGS cells were treated with VacA protein, which was purified from culture supernatants obtained from the *cagA* ^−^/*vacA* ^*s1m1*^ and *cagA* ^−^/*vacA* ^*s1m2*^ *H. pylori* clinical isolates using an affinity chromatography scheme. VacA^*s1m1*^ toxin could induce cell death with PI staining and MTT assay in a time-dependent manner, and VacA^*s1m2*^ toxin did not (Figs. 2a, b). Some studies reported that VacA can induce autophagy in human gastric cancer cells^23–25^. However, whether the activating autophagy promotes or inhibits cell death is unknown. To explore this problem, after pretreatment with a pharmacological inhibitor of autophagy (3-methyladenine; 3-MA) or an apoptosis inhibitor (Z-VAD), AGS cells were treated with VacA^*s1m1/ s1m2*^ toxin, and the level of cell death was subsequently detected by PI staining and MTT assay. 3-MA or Z-VAD could significantly reduce cell death induced by VacA^*s1m1*^ toxin in the AGS cells (Figs. 2c, d). These results suggest that autophagy may have been involved in the VacA-induced cell death.

### VacA toxin induces autophagy in AGS cells

**Fig. 3:**
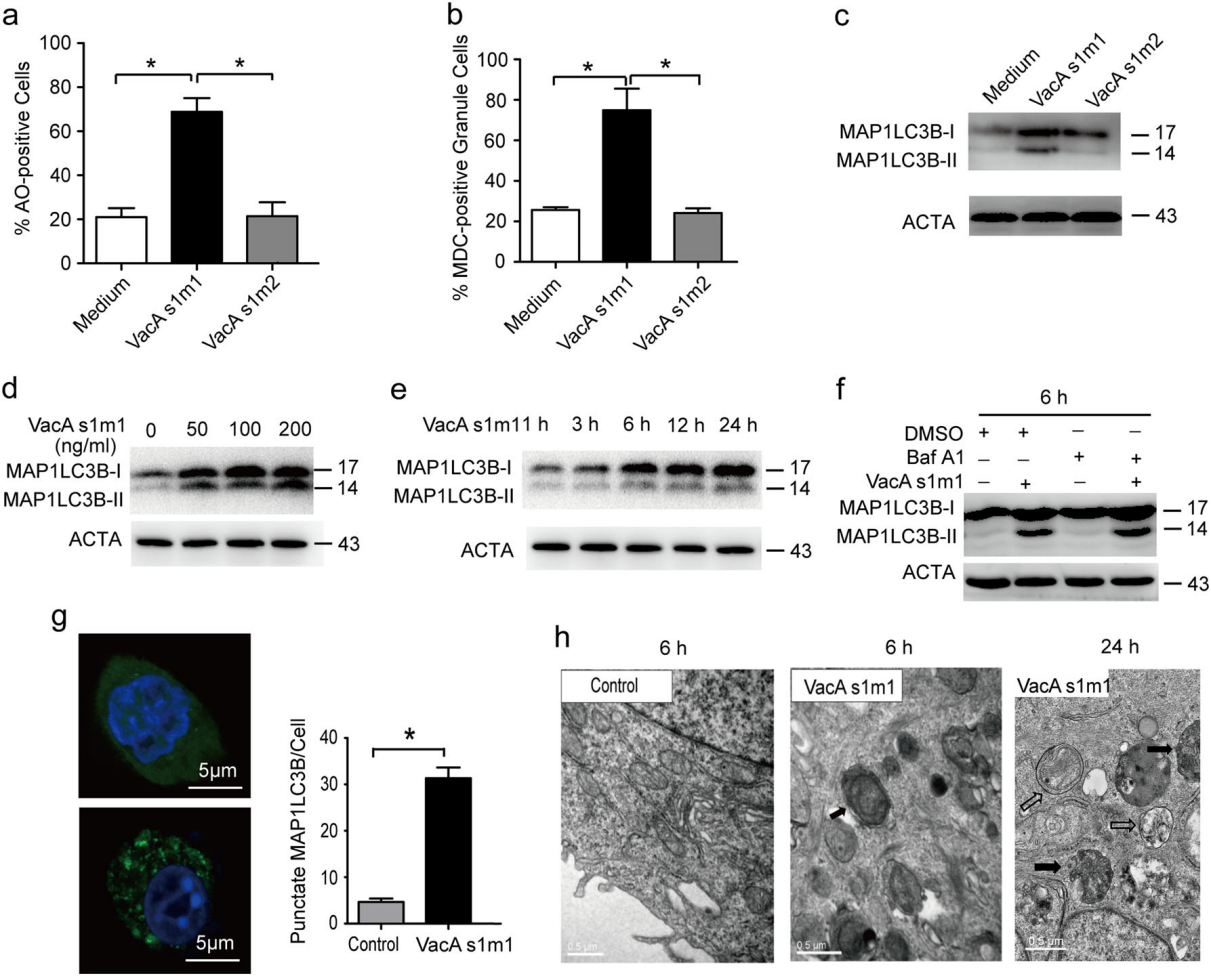
VacA toxin induces autophagy in AGS cells (**a**, **b**) AGS cells were subjected to the indicated treatments for 6 h and were stained with acridine orange or MDC. After incubation, the cells were immediately analyzed by flow cytometry. (**c**) Measurement of the MAP1LC3B-II conversion in AGS cells subjected to the indicated treatments using western blot analysis. (**d**, **e**) VacA increased the conversion of MAP1LC3B-I to MAP1LC3B-II in AGS cells. AGS cells were treated with a gradually increasing concentration of VacA or were treated with 50 ng/ml VacA for different times. (**f**) VacA induced complete autophagic flux in AGS cells. AGS cells were treated with 50 ng/ml VacA for 6 h in the presence of 10 nM Baf A1. (**g**) The number of GFP-MAP1LC3B puncta in each cell was counted using a confocal microscope. (**h**) Representative TEM images of AGS cells treated with VacA treatment for 6 or 24 h. The white arrows indicate the autophagosomes, and the black arrows indicate the autolysosomes. The experiments were performed in triplicate, and all replicates showed similar results. *P < 0.05

To elucidate whether VacA could induce autophagy in AGS cells, we applied flow cytometry to detect the formation of autolysosomes after staining with acridine orange (AO) or monodansylcadaverine (MDC). Intriguingly, VacA^*s1m1*^ toxin induced the percentage of autolysosome-accumulated cells up to more than 70% of AGS cells with the two staining assays (Figs. 3a, b). Compared with VacA^*s1m2*^ and control, VacA^*s1m1*^ significantly increased the number of autolysosomes in AGS cells (Figs. 3a, b). The VacA^*s1m1*^ toxin induced the ratio of microtubule-associated protein 1 light chain 3 beta-II (MAP1LC3B-II) to ACTA (Fig. 3c) and led to MAP1LC3B-II accumulation in time-dependent and dose-dependent manners (Figs. 3d, e). Pharmacological inhibitor of autophagic flux bafilomycin A1 (Baf A1) further promoted the accumulation of MAP1LC3B-II in the AGS cells (Fig. 3f). In addition, GFP-LC3 puncta formation assays and transmission electron microscopy (TEM) assays also showed that VacA^*s1m1*^ could increase the number of autophagosomes in AGS cells after 6 or 24 h (Figs. 3g, h). These data indicate that VacA could induce autophagy in AGS cells.

### ER stress may be involved in VacA-induced cell death

**Fig. 4:**
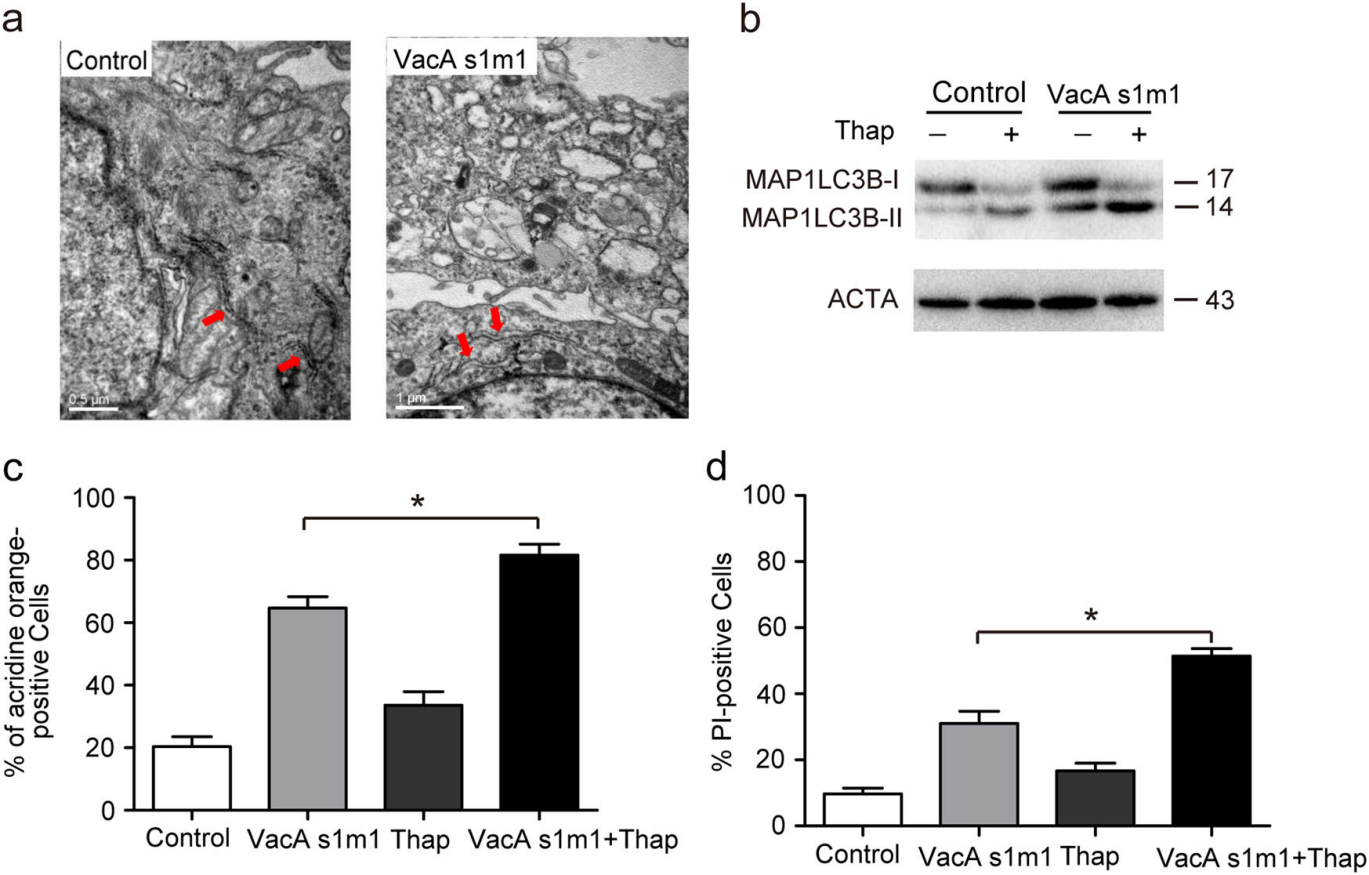
ER stress may be involved in VacA-induced cell death (**a**) The TEM images showed the dilatation of the ER in the VacA-treated cells compared to the untreated cells. The red arrows indicate the ER. (**b**) Measurement of MAP1LC3B-II conversion in AGS cells using western blot analysis. The cells were treated with 50 ng/ml VacA^*s1m1*^ for 6 h in the presence of 200 nM thapsigargin (Thap). (**c**, **d**) AGS cells were exposed to 200 nM Thap, a combination of 200 nM Thap and 50 ng/ml VacA^*s1m*^, or VacA^*s1m1*^ alone for 6 h. The cells that were positive for autophagosomes were detected using an acridine orange staining assay. Cell death was analyzed following PI staining. *P < 0.05

With the exception of autophagosomes in TEM analysis, we also observed numerous cells with dilated ER in VacA^*s1m1*^-treated cells (Fig. 4a). To further determine whether ER stress was involved in VacA^*s1m1*^-induced cell death, an ER stress inducer thapsigargin was applied to confirm the role of ER stress. Western blot analysis showed that thapsigargin could increase the expression of MAP1LC3B-II (Fig. 4b), and the AO staining assay was also consistent with this result (Fig. 4c). Moreover, thapsigargin could increase VacA^*s1m1*^-induced cell death (Fig. 4d). These data showed that ER stress may be involved in VacA-induced cell death.

### VacA induces autophagy through ER stress

**Fig. 5:**
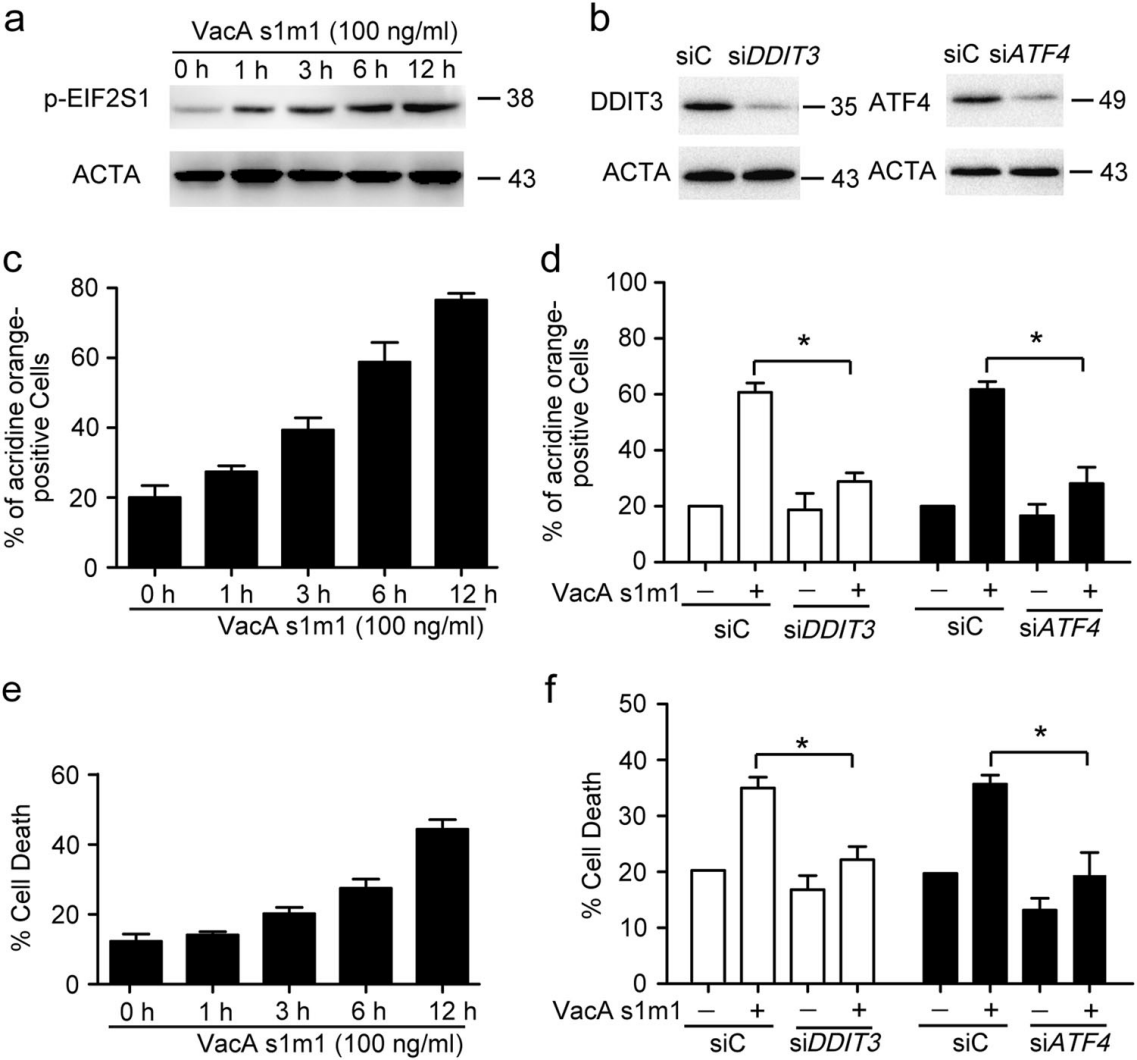
VacA induces autophagy through ER stress (**a**) Measurement of EIF2S1 phosphorylation following 50 ng/ml VacA treatment at different times using western blot analysis. (**b**) The inhibition efficiency of the siRNAs against DDIT3 and ATF4. (**c**, **d**) The quantification of autophagosomes in AGS cells by acridine orange staining. The cells were treated with only 50 ng/ml VacA or were treated with VacA^*s1m1*^ after transfection with siC, siDDIT3, or siATF4 for 24 h. (**e**, **f**) Detection of cell death by PI staining. AGS cells were treated as above. The data shown represent the mean ± SEM of at least three independent experiments. *P < 0.05

To further research the role of ER stress in VacA-induced cell death, the phosphorylation of EIF2S1 (eukaryotic translation initiation factor 2, subunit 1 alpha, 35 kDa) was investigated after VacA^*s1m1*^ treatment. VacA^*s1m1*^ could increase the phosphorylation of EIF2S1 in a time-dependent manner in AGS cells (Fig. 5a). The VacA-induced autophagy and cell death also had the same trend with EIF2S1 phosphorylation (Figs. 5c, e). In addition, knockdown of the ER stress effector DDIT3 or ATF4, two downstream targets of EIF2S1, could drastically decrease the formation of autolysosomes and cell death in the VacA^*s1m1*^-treated AGS cells (Figs. 5b, d, f). To further elucidate the relationship of ER stress to the effects of VacA, mitochondrial dysfunction and apoptosis, we also detected the mitochondrial dysfunction and apoptosis with knockdown of DDIT3 and ATF4 in the VacA-treated AGS cells. VacA could decrease the mitochondrial ATP level and increase apoptosis in a time-dependent manner in AGS cells (Supplementary Figs. 1a, c). Moreover, as shown in Supplementary Figs. 1b, d, knockdown of DDIT3 or TRIB3 could also decrease VacA-induced mitochondrial dysfunction and apoptosis. These results suggest that VacA-induced ER stress is antecedent to autophagy generation.

**Fig. 6:**
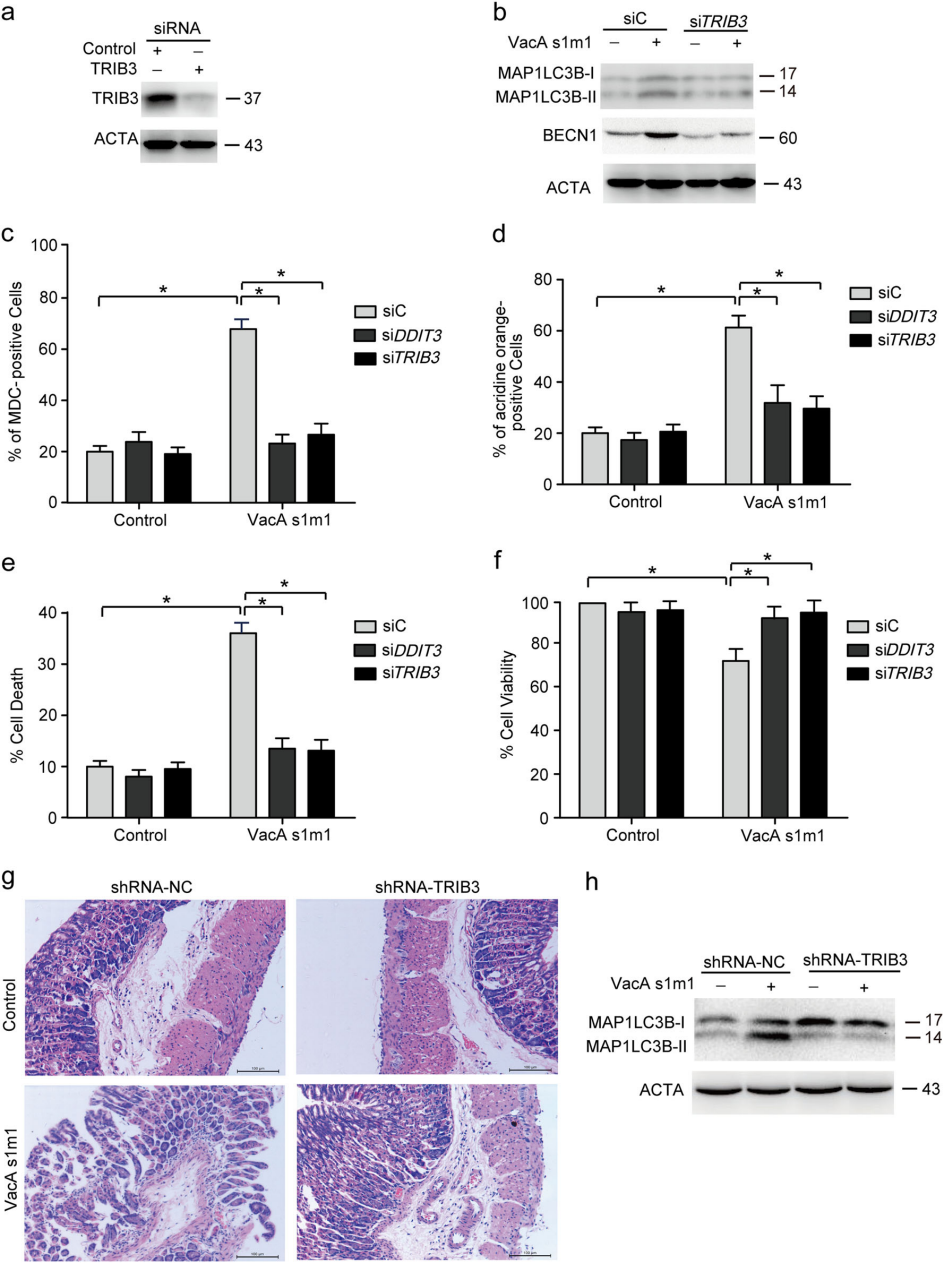
VacA induces autophagy via TRIB3 (**a**) Detection of the inhibition efficiency of siRNAs against TRIB3. AGS cells were transfected with siRNAs targeting TRIB3 (100 nM) for 24 h, and the protein levels of the targets were evaluated using western blot analysis. (**b**) The effect of 50 ng/ml VacA on MAP1LC3B-II conversion and BECN1 in AGS cells transfected with siC or siTRIB3 for 24 h. (**c**–**e**) Detection of MDC, AO, and PI staining of cells that were transfected with siC, siDDIT3, or siTRIB3 using flow cytometry analysis. (**f**) An MTT assay assessed cell viability that AGS cells were transfected with siC, siDDIT3, or siTRIB3 for 24 h. (**g**) Representative images of hematoxylin and eosin staining of gastric epithelium from mouse. The gastric tissues were infected with pGCSIL-GFP-shRNA-TRIB3 or pGCSIL-GFP-shRNA-NC lentivirus for 24 h, and challenged by VacA^*s1m1*^ or the control for 24 h. Scale bar in all panels: 100 μm. (**h**) Measurement of MAP1LC3B-II conversion following TRIB3 shRNA lentivirus or VacA treatment using western blot analysis. The experiments performed in triplicate showed consistent results. *P < 0.05

TRIB3, a target of DDIT3-ATF4, plays an important role in ER stress-induced cell death. To evaluate this role, TRIB3 was knocked down by TRIB3 siRNA in AGS cells (Fig. 6a). Knockdown of DDIT3 or TRIB3 could prevent VacA^*s1m1*^-induced autophagy in AGS cells (Figs. 6b–d). Moreover, knockdown of DDIT3 or TRIB3 could also decrease VacA-induced cell death (Figs. 6e, f). Furthermore, we generated TRIB3 shRNA lentivirus to knockdown the expression of TRIB3 in the gastric tissue of mice. After the tissue was treated with VacA^*s1m1*^ toxin, cell death and autophagy were detected with hematoxylin and eosin staining or western blot analysis. The damage degree of the VacA^*s1m1*^ groups was significantly increased compared to the control, and the damage degree of the shRNA-TRIB3 lentivirus group was lower than the shRNA-NC lentivirus group (Fig. 6g). As shown in Fig. 6h, shRNA-TRIB3 lentivirus could also decrease the ratio of MAP1LC3B-II to ACTA. These data indicated that VacA induces autophagy and cell death in the AGS cells by triggering ER stress, which is involved in the stimulation of EIF2S1 phosphorylation and the upregulation of DDIT3 and TRIB3.

### Autophagy is upstream of apoptosis in VacA-induced cell death

**Fig. 7:**
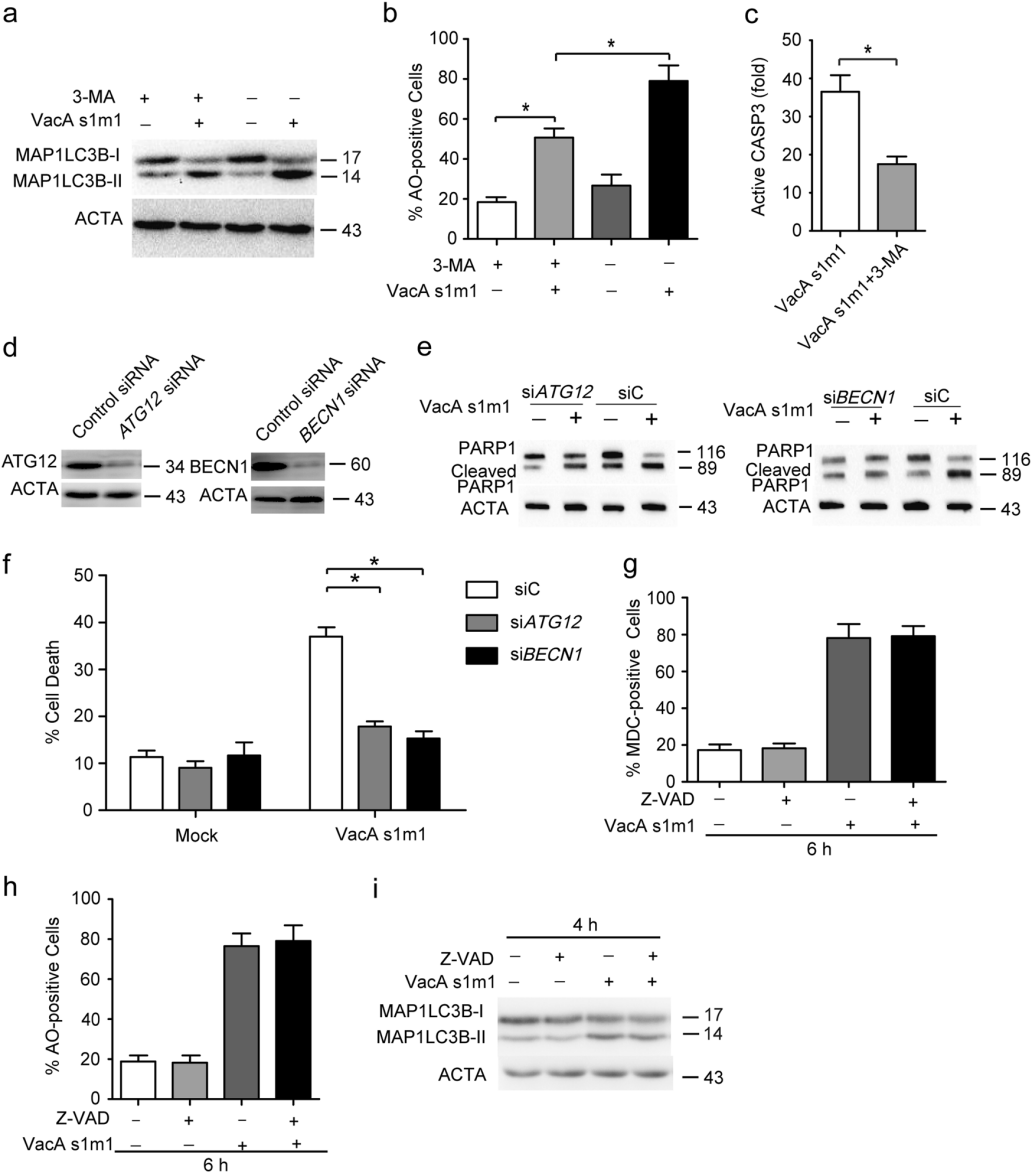
Autophagy is upstream of apoptosis in VacA-induced cell death (**a**) Measurement of MAP1LC3B-II conversion following VacA treatment using western blot analysis. AGS cells were treated with 50 ng/ml VacA for 4 h in the presence of 2 mM 3-MA. (**b**) Detection of AO staining by flow cytometry following VacA treatment. AGS cells were treated as above. (**c**) Detection of active CASP3 following 3-MA pretreatment in cells. (**d**) The inhibition efficiency of siRNAs against ATG12 and BECN1. AGS cells were transfected with siRNAs targeting ATG12 and BECN1 (100 nM each) for 24 h, and the protein levels of the two targets were evaluated using western blot analysis. (**e**) The effect of 50 ng/ml VacA on PARP1 cleavage in AGS cells transfected with siC, siATG12, or siBECN1. (**f**) Detection of cell death by flow cytometry in cells transfected with siC, siATG12, or siBECN1 for 24 h. (**g**, **h**) Detection of MDC and AO staining of cells treated with 50 mM Z-VAD or 50 ng/ml VacA using flow cytometry analysis. (**i**) The MAP1LC3B-II conversion was detected using western blot analysis. AGS cells were treated as above

VacA^*s1m1*^-induced autophagy can be suppressed by 3-MA (Figs. 7a, b). To investigate the relationship of VacA-induced apoptosis and autophagy, autophagy inhibitor 3-MA was applied to analyze active CASP3/caspase 3 in VacA-treated AGS cells. Active CASP3/caspase 3 was decreased by 3-MA (Fig. 7c). To further assess the role of autophagy in VacA-induced cell death, we examined the silencing effect of ATG12 or BECN1 siRNA in AGS cells (Fig. 7d). Knockdown of ATG12 or BECN1 could decrease VacA^*s1m1*^-induced cell death and PARP1 cleavage (Figs. 7e, f). In addition, similar results were also present in Az-521 cells (Supplementary Figs. 2a–c). These results revealed that autophagy generation preceded the appearance of apoptosis by VacA. In addition, to further verify the relationship of autophagy and apoptosis under VacA stress conditions, AGS cells were pretreated with apoptosis inhibitor Z-VAD for 24 h, and autophagy induced by VacA^*s1m1*^ was evaluated with MDC, AO, and western blot assays. Z-VAD had no significant effect on autophagy induced by VacA^*s1m1*^ (Figs. 7g–i). These results suggest that autophagy may be upstream of apoptosis in VacA-induced cell death.

## Discussion

According to the present results, we found that VacA induces autophagic cell death through endoplasmic reticulum stress in human gastric epithelial cells. This novel mechanism is derived from the following results: (i) VacA^*s1m1*^ could significantly increase autophagy and cell death in AGS cells, (ii) the autophagy by VacA^*s1m1*^ is dependent on ER stress, which participates in VacA^*s1m1*^-induced cell death, and (iii) autophagy may be upstream of apoptosis in VacA^*s1m1*^-induced cell death.

VacA exerts pleiotropic actions on gastric cells, including membrane channel formation, vacuolization, immunomodulation, disruption of endosomal/lysosomal function, and apoptosis^26^. Among these cellular activities, cell death induced by VacA plays a critical role in the origination and development of peptic ulcers in *H. pylori* infection. Until now, apoptosis was believed to be a crucial factor in cell death induced by VacA. VacA induces apoptosis by connexin 43 (Cx43) accumulation through a Rac1/ERK-dependent pathway^27^. Although VacA did not affect Cx43 expression, Cx43 accumulation resulted in the apoptotic cell death of AZ-521 cells. Here, we investigated the role of VacA in gastric epithelial cell death through autophagy and ER stress and its underlying molecular mechanism. We demonstrate that VacA induces both autophagy and apoptosis and leads to cell death. Interestingly, autophagy generation appears to occur earlier than apoptosis in AGS cells. Pharmacological inhibitor of autophagy 3-MA or knockdown of ATG12 or BECN1 could inhibit cleavage of PARP, active caspase 3, and cell death (Figs. 7c, e, f). These data indicate that autophagy promotes gastric epithelial cell death in VacA stress conditions and mediates the generation of apoptosis. Although mitochondria are well-known targets of VacA, how VacA affects the mitochondria during apoptosis remains to be elucidated. Recently, one study reported that most VacA is localized to vacuoles, marked by Rab7-GFP, rather than mitochondria using immunostaining and confocal microscopy^10^. Our study reported that VacA could induce ER stress in AGS cells, which is consistent with a previous report in AZ-521 cells^7^. In our study, VacA could increase the phosphorylation of EIF2S1, and knockdown of the ER stress effector DDIT3 or ATF4 could drastically decrease autolysosomes and cell death in the VacA-treated AGS cells. Akazawa et al. also reported that VacA transcriptionally increased C/EBP homologous protein (CHOP) and phosphorylation of EIF2S1 in gastric epithelial cells, and knockdown of CHOP could lead to inhibition of VacA-induced apoptosis^7^. Consistent with the report, our study also indicated that the activation of ER stress mediated by VacA contributes to apoptosis in gastric epithelial cells.

VacA, a critical virulence factor of *H. pylori*, was sufficient to induce autophagy, but prolonged exposure to VacA could also disrupt autophagy in human gastric epithelial cells and lead to infection and cell death. The different effects of VacA on autophagy are dependent on the duration/level of cell exposure to the toxin, but its timing and related molecular mechanisms deserve further investigation. In the current study, our results demonstrated that VacA could induce autophagy in AGS cells. As shown in Fig. 3, VacA significantly induced the ratio of MAP1LC3B-II and the formation of autolysosomes in AGS cells, and an increase in the number of autophagosomes was observed by TEM analysis in the VacA-treated AGS cells. Recently, Yahiro reported that VacA induced Cx43 accumulation and autophagy, and Cx43 colocalized with autophagosomal marker MAP1LC3B-II^28^. Terebiznik et al. found that *H. pylori* triggered autophagy with the appearance of autophagosomes, different in size, and morphology from typical VacA-induced vacuoles^24^. These data indicated that there is a close relationship between VacA and autophagy, and its specific regulatory mechanism will continue to be a fascinating and rewarding subject for future studies.

In summary, these results provide novel evidence that VacA triggers the ER stress response to activate autophagy and induces cell death of AGS cells. Future research involving mouse models may lead to a better understanding of the role of ER stress in the pathogenesis of VacA-induced gastric mucosal injury, and experiments should be designed to establish the dynamics and specific mode of action of VacA in affecting autophagy.

## Materials and methods

### Antibodies and reagents

The adenovirus of the GFP-LC3B fusion protein (C3007) was obtained from Beyotime Institute of Biotechnology. Some chemical reagents were purchased from Sigma, including 3-MA (M9281), Baf A1 (B1793), thapsigargin (T9033), AO (A8097), MDC (30432), and carbobenzoxy-valyl-alanyl-aspartyl-[O-methyl]-fluoromethyl ketone (Z-VAD-FMK, V116); antibodies against autophagy-related protein 12 (ATG12, WH0009140m1), TRIB3 (WH0057761M3), and MAP1LC3B (L7543) were also obtained from Sigma. The antibody against beclin1 (BECN1, 612112) and ACTA (10731) was obtained from BD Transduction Laboratories and Santa Cruz Biotechnology. Other antibodies against poly(ADP-ribose) polymerase 1 (PARP1, 9542), p-EIF2S1 (9721), ATF4 (11815), and DDIT3 (3087) were obtained from Cell Signaling Technology. ATF4 (human, sc-35112), ATG12 (human, sc-72578), BECN1 (human, sc-29797), control siRNA (sc-44230), DDIT3 (human, sc-35437), NUPR1 (human, sc-40792), and TRIB3 siRNAs (human, sc-44426) were obtained from Santa Cruz Biotechnology, and the effect of protein knockdown was evaluated using western blot analysis.

### Cell culture, tissue culture, and bacterial strains

AGS cells and AZ-521 cells, human gastric cancer cell lines, were obtained from the American Type Culture Collection (Manassas, VA, USA). The two cell lines were cultured in F12 cell culture medium (Gibco, Grand Island, NY, USA, #11765-054) or Earle’s minimal essential medium (Sigma, M0275) containing 10% fetal bovine serum (Gibco, #10099-141) and 100 U/mL penicillin/streptomycin (Thermo Scientific, #15140148) in a 5% CO_2_ incubator at 37 °C.

The gastric tissue culture was performed with a modification of the method that was described previously^29^. Briefly, the gastric tissues of C57BL/6 mice were sliced into fragments, followed by soaking in ice-cold PBS (Sigma, P5493) containing gentamicin (0.5 mg/mL; Sigma, G9654), with gentle shaking at 4 °C for 10 min, and then washed three times. The washed gastric tissues were cultured in DMEM medium (Gibco, #11965) containing 10% fetal bovine serum (Gibco, #10099-141) and 100 U/mL penicillin/streptomycin (Thermo Scientific, #15140148) in a 5% CO_2_ incubator at 37 °C. Finally, the tissues were infected with pGCSIL-GFP-shRNA-TRIB3 or pGCSIL-GFP-shRNA-NC (2 × 10^8^ TU/mL) for 24 h and then challenged with VacA^s1m1^ toxin or the control for 24 h. Histological assessment was performed according to the Sydney classification by two pathologists who were blinded to the other experimental results. The study was approved by the ethics review board at Third Military Medical University.

The *cagA* ^−^/*vacA* ^*s1m*^ and *cagA* ^−^/*vacA* ^*s1m2*^ *H. pylori* clinical isolates were obtained from Southwest Hospital in Chongqing. The brain-heart infusion plates containing 10% rabbit blood were used to cultivate these *H. pylori* clinical isolates at 37 °C under microaerophilic conditions (85% N_2_, 10% CO_2_, and 5% O_2_). In patients with positive culture, *H. pylori* isolates were subcultured for a maximum of five passages, and genomic DNA was extracted to genotype the cagA and vacA genes, as previously described^30^. The primers used for PCR amplification and nucleotide sequencing are listed as follows: primers for CagA, forward: 5′-GAGTCATAATGGCATAGAACCTGAA-3′, reverse: 5′-TTGTGCAAGAAATTCCATGAAA-3′; primers for VacA-s, forward: 5′-ATGGAAATACAACAAACACAC-3′, reverse: 5′-CTGCTTGAATGCGCCAAAC-3′; primers for VacA-m, forward: 5′-CAATCTGTCCAATCAAGCGAG-3′, reverse: 5′-GATAACAGCCAAGCTTTTGAGG-3′.

### Purification of VacA

VacA was purified from culture supernatants obtained from the *cagA* ^−^/*vacA* ^*s1m1*^ and *cagA* ^−^/*vacA* ^*s1m2*^ *H. pylori* clinical isolates using an affinity chromatography scheme. The immunoaffinity chromatography column was prepared by VacA subunit-specific monoclonal antibody 5E4 (Santa Cruz, 32746) coupled to CNBr-activated sepharose 4B matrix (GE Healthcare, 17043001). Then, an affinity chromatography column (GE Healthcare, 17040401) was used to purify the samples containing VacA. The relative molecular weight of VacA was 88 kDa, confirmed by SDS-PAGE.

### TRIB3 shRNA lentivirus generation

Inverted and self-complementary hairpin DNA oligos targeting mouse TRIB3 mRNA (GeneBank No. NM-175093.2) were obtained from Genchem Biotechnology Company (Shanghai, China), and the sequences were as follows: sense, 5′-CCGGGAAGAAACCGTTGGAGTTTGATTCAAGAGATCAAACTCCAACGGTTTCTTCTTTTTG-3′ and antisense, 5′-AATTCAAAAAGAAGAAACCGTTGGAGTTTGATCTCTTGAATCAAACTCCAACGGTTTCTTC-3′. The negative control sequences, which have been used in a number of studies, were as follows: sense, 5′-CCGGTTCTCCGAACGTGTCACGTTTCAAGAGAACGTGACACGTTCGGAGAATTTTTG-3′ and antisense, 5′-AATTCAAAAATTCTCCGAACGTGTCACGTTCTCTTGAAACGTGACACGTTCGGAGAA-3′, including negative control scrambled shRNA pair sequences, which had no significant homology to any mouse gene sequences.

The pGCSIL-GFP vector was digested by AgeI and EcoRI and purified by Qiagen (Shanghai, China), a quick gel extraction kit. The pairs of complementary hairpin DNA oligos that were mentioned above were synthesized, annealed, and ligated into linearized pGCSIL-GFP vector. The ligated DNA solution was transformed into *E. coli* DH5α and incubated on a Luria Bertani plate (50 ng/ml ampicillin) at 37 °C for 16 h. Positive clones were identified by DNA sequence analysis, and the resulting plasmids were called pGCSIL-GFP-shRNA-TRIB3 and pGCSIL-GFP-shRNA-NC. Lentiviruses were generated in 293T cells by co-transfection of pGCSIL-GFP-shRNA-TRIB3 or pGCSIL-GFP-shRNA-NC with pHelper1.0 and pHelper2.0 plasmids. These plasmids were transfected into 70% confluent 293T cells using Lipofectamine 2000 (Invitrogen). Then, lentiviral particles were harvested from the media 48 h after transfection and purified with ultracentrifugation. The final concentration of the virus suspension was 2 × 10^12^ TU/L.

### Transmission electron microscopy

AGS cells were treated with 50 ng/ml VacA^*s1m1*^ toxin for 6 h, collected, and fixed in a glutaraldehyde and sodium cacodylate solution for 2 h, and then fixed with 1% OsO_4_ for 1 h and a half, and then stained in 3% aqueous uranyl acetate for 1 h. These cells were dehydrated with graded alcohol (50%, 70%, 80%, 90%, 95%, 100%) and embedded in Epon-Araldite resin (Canemco, #034). Ultrathin sections were prepared on a Leica EM UC7 ultramicrotome, counterstained with 0.3% lead citrate, and observed under a Zeiss EM 902A electron microscope. The autophagosome counting method was followed as described previously by Yla-Anttila et al^31^.

### ATP measurements

After treatment with 100 nM siRNA or 50 ng/ml VacA^*s1m1*^ toxin, cells were collected, centrifuged, and washed with phosphate-buffered saline (PBS) three times. Next, 200 μL lysis buffer from an ATP Assay kit (Beyotime, S0026) was added to each tube and then ultrasonicated. The lysates were centrifuged at 12,000 r.p.m. for 5 min at 4 °C. The supernatant was transferred to a new 1.5-mL tube for the ATP assay with the ATP detection kit. The protein level of the supernatant was measured at 562 nm with Bicinchoninic Acid assay (Beyotime). The relative ATP level was calculated according to the following formula: relative ATP level = ATP value/protein value.

### GFP-LC3 puncta formation assays

AGS cells were infected with GFP-LC3B adenovirus (MOI = 100:1) for 24 h, then cultured with 50 ng/ml VacA^*s1m1*^ toxin for 6 h, and then fixed in 4% paraformaldehyde for 10 min at 37 °C. Confocal microscopy was performed with a Radiance 2000 laser scanning confocal microscope (Bio-Rad, San Francisco, CA), followed by image analysis with LaserSharp 2000 software (Bio-Rad). Images were acquired in a sequential scanning mode. According to methods for monitoring GFP-LC3 puncta formation assays, the average number of MAP1LC3B puncta per cell in GFP-MAP1LC3B-positive cells was determined.

### Western blot analysis

Western blotting was performed to determine the protein level of ATG12, MAP1LC3B, p-EIF2S1, ATF4, TRIB3, PARP1, DDIT3, BECN1, and ACTA in AGS cells as described previously^17^. AGS cells were washed and then lysed by the M-PER Mammalian Protein Extraction Reagent (Pierce, 78501, Thermo Scientific, Waltham, MA, USA). After centrifugation, the total protein concentration was determined with a BCA protein assay kit (Pierce, 23227, Thermo Scientific). Cell lysis solutions were separated using SDS-PAGE and transferred to polyvinylidene difluoride (PVDF) membranes. Primary antibodies were diluted 1:1000. Membranes were developed using Supersignal® West Dura Duration substrate reagent (Thermo Scientific, 34080). Densitometric analysis on the western blot was performed by Image Gauge software (Fujifilm, MD, USA). ACTA was used as an internal control, and the ratio of the intensity of the protein of interest to ACTA was calculated.

### Cell viability

According to the manufacturer’s instructions (Sigma), AGS cell viability was determined with an MTT assay. Following treatment, AGS cells were incubated with MTT at a final concentration of 5 mg/L for 2 h and then dissolved in the MTT solubilization solution. The cell survival rate was measured with an absorbance at 590 nm (A590) by a microplate reader (Bio-Rad).

### MDC staining and AO staining assays

Following treatment with *H. pylori* or transfection with plasmids/siRNAs, AGS cells were stained with 10 mM MDC at 37 °C for 10 min and fixed in 3% paraformaldehyde for 30 min. A Radiance 2000 laser scanning confocal microscope was used to take photographs. Other cells with the same treatment were quantified by flow cytometry (BD FACScan Flow cytometer).

In AO staining assays, AGS cells were stained with 1 mg/mL AO solution for 15 min at 37 °C. After incubation, cells were washed and immediately analyzed with a Radiance 2000 laser scanning confocal microscope. Then, other cells with the same treatment were quantified by flow cytometry.

### Cell death assay

AGS cell death was assessed using a PI staining assay as previously described^17^. The cells were trypsinized, collected, and resuspended in 2 ml of PBS, and then incubated with the PI staining solution at 37 °C for 30 min in the dark before being finally measured with flow cytometry.

### Apoptosis

The ratio of apoptotic cells was evaluated by staining 5 × 10^5^ cells with an ANXA5/annexin V-FITC/PI Detection Kit (Invitrogen, V13242) according to the manufacturer’s protocol^17^. The samples were analyzed by flow cytometry (BD FACScan Flow cytometer, USA) to determine the percentage of cells displaying annexin V^+^/PI^−^ (early apoptosis) or annexin V^+^/PI^+^ staining (late apoptosis). For each sample, we report the percentage values corresponding to annexin V-FITC-positive cells. Three independent experiments were performed for each assay condition.

### Statistical analysis

The results are expressed as the mean ± SEM. Two group data were analyzed by Student’s *t*-test, and multiple group data were analyzed using one-way ANOVA. Statistically significant differences are indicated by asterisks (*P < 0.05, **P < 0.01).

## Electronic supplementary material

Supplementary Materials
