# Supplementary material for: Helicobacter pylori VacA induces autophagic cell death in gastric epithelial cells via the endoplasmic reticulum stress pathway
Source: Cell Death Dis. 2017 Dec 13;8(12):3207. doi: 10.1038/s41419-017-0011-x (PMC5870595; doi:10.1038/s41419-017-0011-x)
Supplement: Supplementary file 1 — Supplementary Materials [file 41419_2017_11_MOESM1_ESM.doc]

**Supplementary Information**


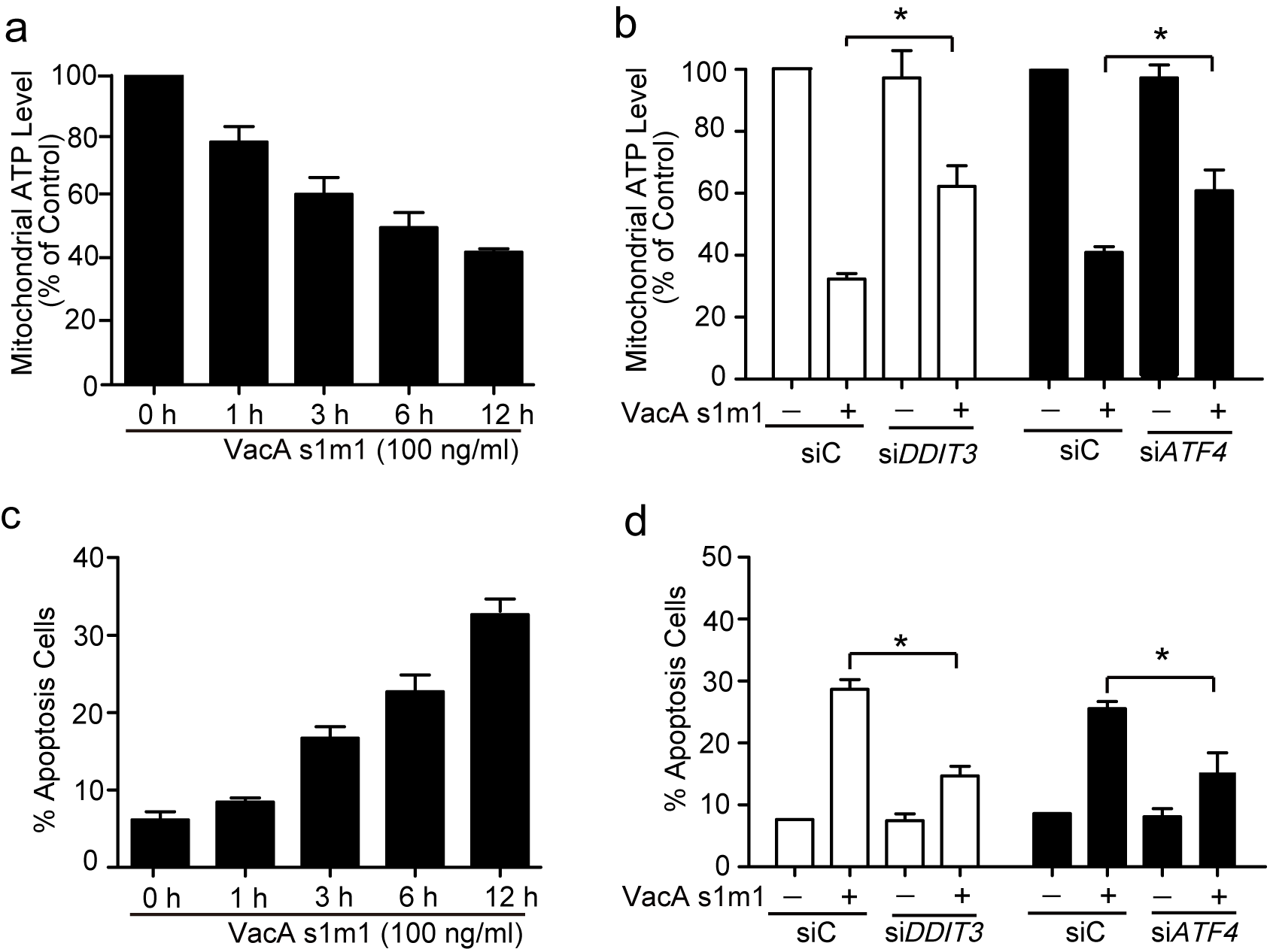


**Supplementary Figure 1** (**a**, **c**) VacA *^s1m1^* toxin treatment reduced ATP production, and increased apoptosis in the indicated time. (**b**, **d**) siDDIT3 and siATF4 increased ATP production, and decreased apoptosis after treatment of VacA *^s1m1^* toxin in AGS cells. * *P* < 0.05.


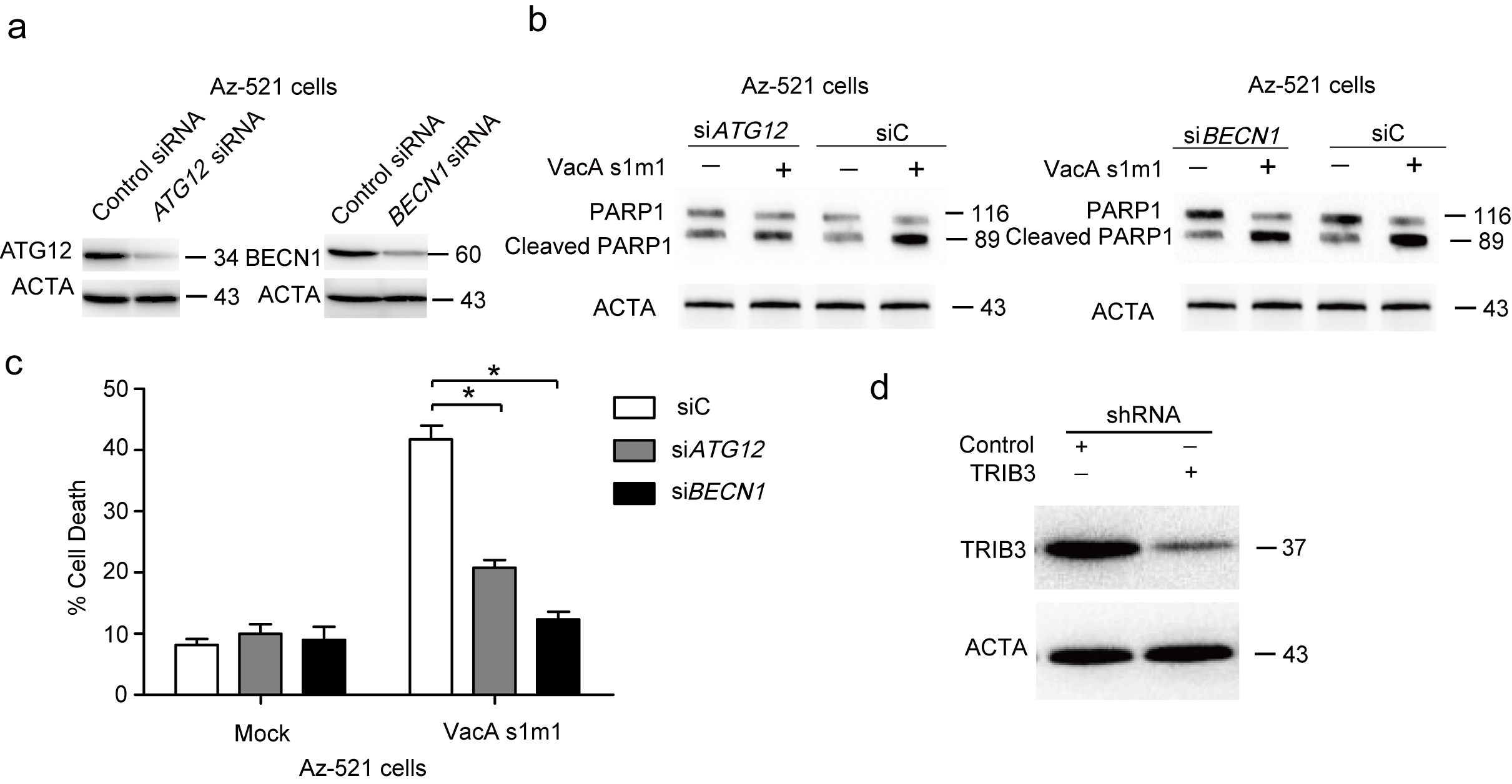


**Supplementary Figure 2** (**a**) The inhibition efficiency of siRNAs against ATG12 and BECN1. Az-521 cells were transfected with siRNAs targeting ATG12 and BECN1 (100 nM each) for 24 h, and the protein levels of the 2 targets were evaluated using western blot analysis. (**b**) The effect of 50 ng/ml VacA on PARP1 cleavage in Az-521 cells transfected with siC, siATG12, or siBECN1. (**c**) Detection of cell death by flow cytometry in cells transfected with siC, siATG12, or siBECN1 for 24 h. (**d**) The inhibition efficiency of shRNA against TRIB3. Isolated gastric tissue mass of mouse were transfected with shRNAs targeting TRIB3 (2×10^8^ TU/ml) for 24 h, and the protein levels of TRIB3 were evaluated using western blot analysis. * *P* < 0.05.
